# Supplementary material for: Evidence supported by Mendelian randomization: impact on inflammatory factors in knee osteoarthritis
Source: Front Med (Lausanne). 2024 May 28;11:1382836. doi: 10.3389/fmed.2024.1382836 (PMC11165061; doi:10.3389/fmed.2024.1382836)
Supplement: Supplementary file 1 [file Data_Sheet_1.ZIP › data/GCST90274787_5e6/787.forest.pdf]

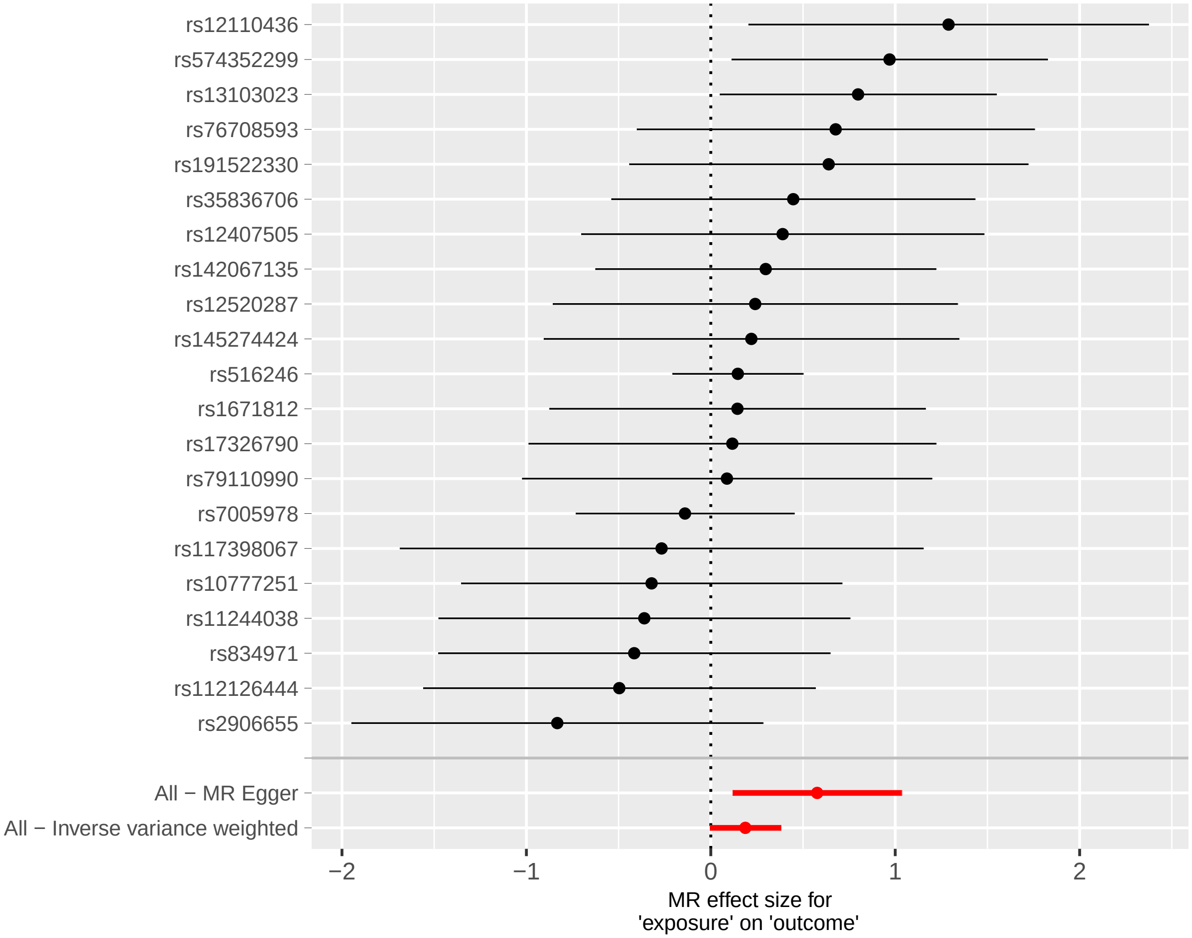
rs12110436
rs574352299
rs13103023
rs76708593
rs191522330
rs35836706
rs12407505
rs142067135
rs12520287
rs145274424
rs516246
rs1671812
rs17326790
rs79110990
rs7005978
rs117398067
rs10777251
rs11244038
rs834971
rs112126444
rs2906655
All − MR Egger
All − Inverse variance weighted
−2
−1
0
1
2
MR effect size for
'exposure' on 'outcome'
